# Fetal Cardiac Doppler Signal Processing Techniques: Challenges and Future Research Directions

**DOI:** 10.3389/fbioe.2017.00082

**Published:** 2017-12-22

**Authors:** Saeed Abdulrahman Alnuaimi, Shihab Jimaa, Ahsan H. Khandoker

**Affiliations:** ^1^Department of Electrical and Computer Engineering, Khalifa University, Abu Dhabi, United Arab Emirates; ^2^Department of Biomedical Engineering, Khalifa University, Abu Dhabi, United Arab Emirates

**Keywords:** fetal Doppler, signal processing, fetal cardiac intervals, fetal monitoring, fetal heart rate, fetal cardiology

## Abstract

The fetal Doppler Ultrasound (DUS) is commonly used for monitoring fetal heart rate and can also be used for identifying the event timings of fetal cardiac valve motions. In early-stage fetuses, the detected Doppler signal suffers from noise and signal loss due to the fetal movements and changing fetal location during the measurement procedure. The fetal cardiac intervals, which can be estimated by measuring the fetal cardiac event timings, are the most important markers of fetal development and well-being. To advance DUS-based fetal monitoring methods, several powerful and well-advanced signal processing and machine learning methods have recently been developed. This review provides an overview of the existing techniques used in fetal cardiac activity monitoring and a comprehensive survey on fetal cardiac Doppler signal processing frameworks. The review is structured with a focus on their shortcomings and advantages, which helps in understanding fetal Doppler cardiogram signal processing methods and the related Doppler signal analysis procedures by providing valuable clinical information. Finally, a set of recommendations are suggested for future research directions and the use of fetal cardiac Doppler signal analysis, processing, and modeling to address the underlying challenges.

## Introduction

Fetal heart rate (FHR) monitoring has been extensively used to assess fetal well-being. The process of FHR monitoring is commonly used during prenatal screening to detect possible fetal health problems that may result in neurological damage or in some cases fetal death during labor. Statistics have shown that 1 out of every 125 babies is born with some kind of congenital cardiac defect (Anisha et al., 2014). When certain pregnancy risk factors have been identified, the FHR must be monitored during labor as a routine physiological measurement (Elmansouri et al., 2014). Cardiotocography (CTG) is the standard methodology in hospitals to monitor fetal well-being and is based on the recording of FHR using a special device. This methodology is popular and commonly used because it is easy to use, is a non-invasive technique, has no contraindications, and can be used frequently. The main disadvantage of CTG is its high sensitivity to fetal movement, as the detection of FHR mostly relies on the correct positioning of the ultrasound probe. This probe therefore needs to be adjusted in the case of fetal movement to afford accurate measurements. Furthermore, the Doppler ultrasound (DUS) transducer is uncomfortable, and the FHR monitoring procedure involves sending a 2 MHz signal toward the fetus. Consequently, it is considered an invasive method and it is not recommended, especially for recordings over long periods under severe conditions (Maeda, 1990; Elmansouri et al., 2014).

In this review paper, the fetal electrocardiogram (FECG) is mentioned many times as a reference to compare and verify the results of the latest fetal cardiac Doppler signal processing techniques. The FECG carries essential information about the fetal heart function. The characteristics or features of the FECG are vital for revealing the fetal development, as well as the existence of fetal distress or congenital heart defects. The FECG is considered to be an effective tool for diagnosing specific structural defects. Currently, there are two methods for recording the FECG: indirect and direct measurements. ST analysis is considered to be a direct method and is performed by directly attaching an electrode to the scalp of the fetus to provide a clean ECG signal. However, this method is not used extensively because of its inherent danger to both the mother and fetus and it can be used in clinical practice only after 36 weeks of gestation. Indirect measurement involves non-invasive FECG recordings that are obtained by placing a skin electrode on the mother’s abdomen. Fetal monitoring is based entirely on the FHR because there is no available technology to reliably measure FECG (Chandraharan, 2010; Anisha et al., 2014; Bsoul, 2015).

Most fetal cardiac defects have some variation in their morphology, which reflects the health status of the fetus, although morphological analysis is lacking in other conventional methods. Most of the clinically essential data in the FECG signal are embedded in the amplitude and duration of its waveforms. Fetal cardiac waveforms help doctors to diagnose fetal arrhythmias such as bradycardia, tachycardia, asphyxia, and congenital heart disease (Voicu et al., 2010; Anisha et al., 2014).

This paper is organized as follows: Section “Introduction” discusses the principles of Doppler-based fetal monitoring, the challenges of fetal cardiac Doppler monitoring, and its biomedical applications. Section “Fetal Heart Rate Monitoring” provides an overview about FHR monitoring and a comprehensive survey of the fetal cardiac Doppler methods based on signal processing techniques. Section “Future Work” provides a summary of the existing challenges and suggests potential directions for future research.

### Fetal Cardiac Doppler Ultrasound

**Figure 1:**
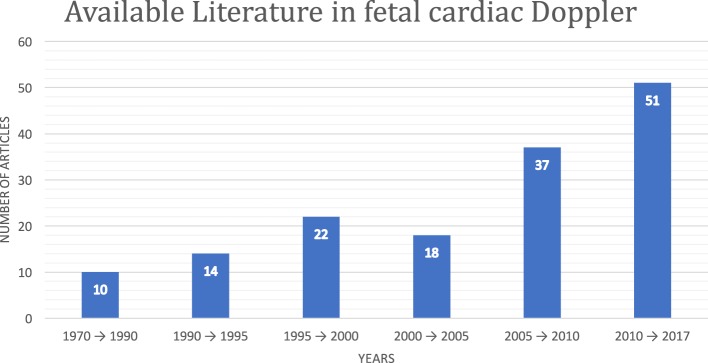
Available fetal cardiac DUS literature in the Institute of Electrical and Electronics Engineers, ScienceDirect, and PubMed databases.

**Figure 2:**
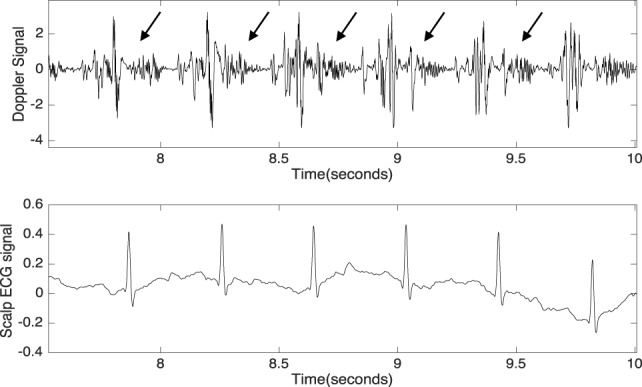
Simultaneously captured DUS and fetal electrocardiogram signals over a period of 2.5 s. The arrows indicate the high-frequency component of the DUS signal.

Although fetal DUS was discovered many years ago, research interest in this field has only arisen over the last few years. Figure 1 shows the number of articles published in the Institute of Electrical and Electronics Engineers (IEEE), ScienceDirect, and the National Institutes of Health (PubMed) databases. The search was focused on both fetal cardiac Doppler and CTG specifically. Figure 2 shows a simultaneously captured fetal cardiac DUS and FECG. The high-frequency component of the DUS signal allows the manual identification of the fetal cardiac valve movements. These sensitive markers can be used for assessing the fetal cardiac performance (Merz, 2004; Marzbanrad et al., 2014).

The form of the fetal cardiac Doppler signal changes over time, as a result of the changes in the transducer location in relation to the moving signal source. This non-stationary nature results in changes in the fetal cardiac Doppler signal on a beat-to-beat basis, in addition to alteration of the spectral characteristics and signal content over time. The resulting complexity makes precise measurement of time intervals in the cardiac cycle quite challenging. The accuracy of calculating the FHR using DUS is very low, and the manual identification of beat-to-beat opening and closing of valves is time consuming, requires special expertise, and is subject to inter- and intra-observer and visual errors. This challenge can be solved by the automated identification of valve movement. Furthermore, DUS signal quality is crucial for the reliable estimation of the fetal cardiac valve timings and also to provide real-time feedback to the operator during data collection; this issue can be solved using automated DUS quality assessment (Wrobel, 2004; Marzbanrad et al., 2015a, 2016).

### Biomedical Applications of Doppler Ultrasound

#### Echocardiography

An echocardiogram, often known as a cardiac echo, is a sonogram of the heart. It allows the visualization of the internal structure of the fetal heart by creating images using the standard 2D, 3D, and Doppler ultrasound. This technique is used routinely during pregnancy in the diagnosis, management, and follow-up of pregnant women carrying a fetus with any suspected or known heart abnormality. This advanced method can provide a significant amount of helpful information, such as pumping capacity, the shape of the heart, and the presence and location of any cardiac tissue damage. Furthermore, it can also allow physicians and researchers to perform a wealth of helpful calculations to estimate the heart function, including the calculation of the cardiac output and heart diastolic function, which indicates how well the heart relaxes.

The most important advantage of echocardiography is that it is a non-invasive technique without any major side effects, except being exposed many times to its high-frequency sound waves which might harm the patient. Pulsed-wave Doppler ultrasound can be used to generate an accurate assessment of the blood flowing through the heart. To visualize any abnormalities, color Doppler and spectral Doppler can be used. Moreover, these methods can be used for discovering any valve-related issues, such as blood leaking through the valves and estimating how well the valves open (Kwon and Park, 2016; Michelfelder et al., 2016).

#### Doppler Fetal Monitor

In 1964, Callagan developed the Doppler ultrasound monitor. A Doppler FHR monitor is a handheld ultrasound transducer used to detect the fetal heart beat during prenatal care. This device uses the Doppler effect to provide an audible simulation of the heartbeat. The use of this monitor is sometimes known as Doppler auscultation. To enhance the sonography, Doppler measurements can be made by employing the Doppler effect to assess the movement of the cardiac structures toward or away from the probe and the corresponding velocity of this movement. For example, the speed and direction of the blood flow in a fetal heart valve can be determined and visualized by calculating the frequency shift of a particular sample volume. The current advanced ultrasound transducers use pulsed Doppler ultrasound for the velocity measurements. The pulsed-wave scanners operate by transmitting and receiving a series of pulses, and the frequency shift can be obtained using the relative phase changes of the pulses. The most important advantage of pulsed Doppler over continuous-wave Doppler is that it permits distance information to be obtained and gain correction to be applied. However, the disadvantage is that the Doppler measurements can be affected by aliasing (Feinstein et al., 1993; Jezewski et al., 2008).

## FHR Monitoring

Fetal heart rate monitoring provides important information regarding fetal cardiac conditions and is a standard clinical procedure that is widely used during pregnancy and labor to assess the well-being of the fetus. FHR monitoring has become a common clinical practice since its introduction at Yale University in 1958. FHR tracing can be performed automatically using electrocardiograms and CTG, whereas FHR monitoring and its interpretation are conducted manually by obstetricians, which can lead to substantial inter-observer and intra-observer differences. In order to allow accurate and consistent clinical practice, automated FHR monitoring and interpretation is recommended (Hon, 1958).

A number of methods have been developed to address various issues in computerized automatic FHR monitoring and interpretation, including FHR signal modeling and representation, feature extraction, and pattern classification. In FHR monitoring devices, the Doppler effect is the preferred technique. Furthermore, a reliable fetal heart simulator becomes essential for testing Doppler FHR monitoring devices (Liu et al., 2008).

### Developing a Doppler FHR Testing Device and Generating the Doppler Frequency Shift

A number of authors have designed a device that can simulate the fetal heart valves and cardiac wall motion in air. This device can be used to test Doppler FHR monitoring in a clinical environment by using a modified electrical relay in air and generating a similar Doppler frequency shift to the fetal heart activity. Accordingly, by modifying the opening and closing velocities of the relay, similar Doppler frequency shifts to the fetal heart activity in soft tissue detected by the ultrasound probe can be produced. The authors tested the functionality and accuracy of the new device with current commercially available Doppler FHR monitors by focusing on the effect of the relay on the frequency range of the Doppler shift. A band-pass filter was used to eliminate the effects of fetal breathing movement, hiccup movement, global movement, and maternal activity, to allow calculation of the periodicity of the frequency reflected by fetal cardiac activity. The authors provided a comparison between the Doppler frequency shifts of this device and the measurements from previous studies and its applied filters to describe the Doppler frequency shift by fetal cardiac activity given in Table S3 in Supplementary Material (Mert et al., 2015).

After comparing the estimated Doppler frequency shifts of the new device with the previously proposed techniques, both the Doppler shift of the fetal cardiac valve and the wall in the same period could be simulated by determining the difference between the opening and closing velocities of the relay armature. The proposed method was tested with commercially available Doppler FHR monitors and the authors claimed that only 4 tests out of 10 displayed a trivial error. Therefore, the proposed device functionality and precision have been proved, demonstrating the potential accuracy of Doppler FHR monitoring devices and the possibility of avoiding any errors in the clinical diagnosis procedures. The authors also claimed that the easy and low-cost implementation could make this device a good candidate for further applications in the future (Mert et al., 2015).

### Real-Time Signal Processing for FHR Monitoring

A new algorithm has been developed based on adaptive thresholding, differencing of local maxima and minima, digital filtering, and statistical properties in the time domain. This algorithm can be used to simultaneously measure the FHR and maternal heart rate from the maternal abdominal electrocardiogram during both pregnancy and labor for ambulatory monitoring. The researchers used a microcontroller-based system to implement the proposed algorithm in the real-time domain. For statistical comparison, a Doppler ultrasound fetal monitor was used on five volunteers with low-risk pregnancies at a gestational age of 35–40 weeks. The proposed algorithm was reported to deliver an average percent root-mean-square difference of 5.32% and a linear correlation coefficient of 0.84–0.93 (Ibrahimy et al., 2003). Furthermore, the FHR curves remained inside a ±5 beats per minute limit relative to the reference ultrasound method for 84.1% of the time (Ibrahimy et al., 2003).

### A Novel Technique for FHR Estimation from the DUS Signal

A new method was recently proposed based on providing accurate beat-to-beat cardiac cycle values of the FHR through multiple measurements of a given fetal cardiac cycle in the DUS signal. The proposed algorithm involves three stages: (i) the dynamic adjustment of the autocorrelation window, (ii) the adaptive autocorrelation Doppler signal peak detection, and (iii) the determination of beat-to-beat time durations. The researchers compared the estimated FHR values and the calculated indices describing the variability of FHR to the reference data obtained from the direct fetal ECG, as well as to another method for FHR estimation. The authors claimed that the results showed that the proposed method increases the estimation precision in comparison to the conventionally used FHR monitoring devices, and this enabled them to calculate reliable parameters describing the FHR variability. Furthermore, according to a comparison of the results of the proposed method to those of the other FHR estimation methods applied, the former technique rejected a much lower number of measured cardiac cycles as being unacceptable (Jezewski et al., 2011).

The proposed technique for the FHR estimation on a beat-to-beat basis provided a high accuracy for the fetal heart interval measurement and enabled a reliable, accurate, and quantitative assessment of the FHR variability, while reducing the number of invalid cardiac cycle measurements. Comparing the proposed method with Peters’ method (CHL et al., 2004), the authors noted a very similar accuracy for the measured intervals and slightly superior findings in terms of the variability indices of the single-measurement method (mean relative error: −5.1%). The authors also mentioned the requirement for assessing the short-term variability for reliable evaluation of FHR signals because it does not depend directly on the accuracy of the fetal cardiac interval measurement. However, in the case of both the proposed method and Peters’ method, the mean relative errors of the short-term variability did not exceed −7%, which might be a satisfactory result (Jezewski et al., 2011).

### Fetal Cardiac Systolic Time Intervals (STIs)

Several antenatal fetal cardiac assessment techniques have been developed to evaluate antepartum fetal cardiac risks. These represent sensitive markers for assessing fetal cardiac performance and permit the evaluation of the fetal cardiac electromechanical coupling. This evaluation process is a fundamental and clinically significant aspect of determining the heart physiology (Weissler et al., 1968; Lewis et al., 1977; Marzbanrad et al., 2014, 2015a).

**Figure 3:**
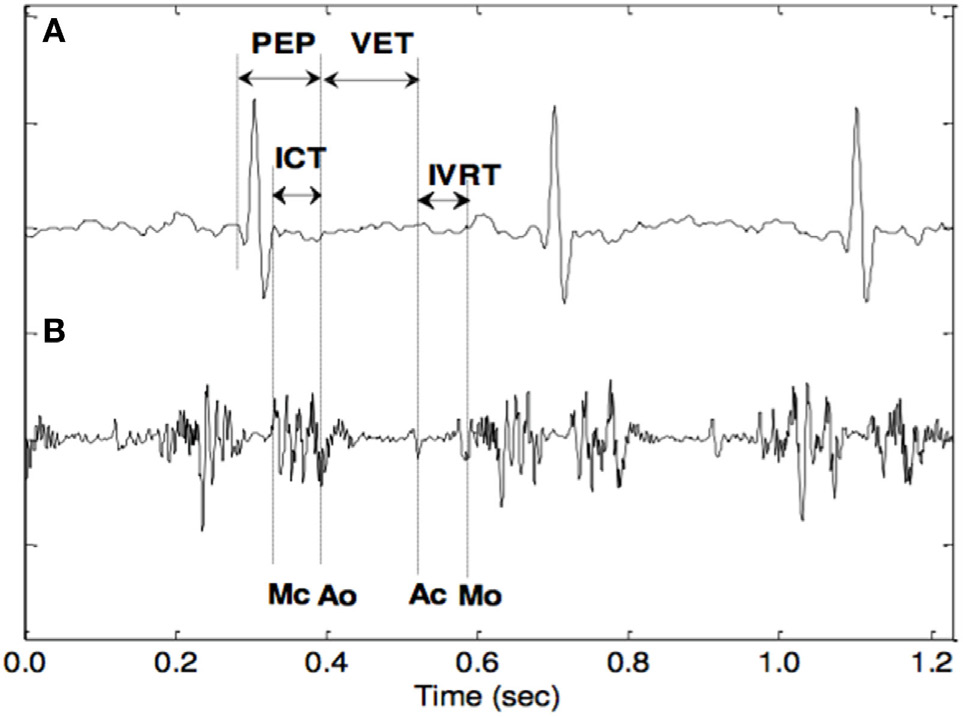
The opening and closing timings of the fetal aortic and mitral valves in relation to the fetal electrocardiogram (Khandoker et al., 2010).

The main basis for estimating these electromechanical markers are the opening and closing timings of the fetal cardiac valves. Figure 3 shows the STIs, which are one of the markers that have received considerable attention as an indicator of myocardial function. From a clinical perspective, the most convenient of the STIs are the pre-ejection period (PEP), the isovolumetric contraction time (ICT), and the left ventricular ejection time. The PEP is a sensitive sign of the functional state of the fetal myocardium and becomes prolonged early in the development of hypoxemia and acidosis. The ICT has also been suggested as a reliable index to represent fetal cardiac contractility. To obtain and analyze the STIs, several non-invasive methods have been proposed such as DUS and abdominal ECG. The cardiac timings were identified manually after filtering the DUS signal using a band-pass filter. The poor quality of the abdominal ECG and the high variability of the fetal cardiac Doppler signal over time are the main limitations of this technique (Murata and Martin, 1974; Murata et al., 1978; Koga et al., 2001; Shakespeare et al., 2001; Yumoto et al., 2005; Marzbanrad et al., 2014).

The DUS signal was divided into different frequency shift ranges by using a digital narrow band-pass filter. From the peaks in one of the filtered signals, the mitral and aortic valve motions were identified. In contrast, other researchers used the short-time Fourier transform method (STFT) to analyze the DUS signal. Correspondingly, the high-frequency component of the DUS signal is related to the valve movements, whereas the low-frequency component is linked with the cardiac wall motion (Koga et al., 2001; Shakespeare et al., 2001).

### The DUS Signal Multiresolution Wavelet Analysis

In another study (Khandoker et al., 2009a), the multiresolution wavelet analysis technique was applied to the fetal cardiac Doppler signal (Marzbanrad et al., 2016). Wavelet analysis is a powerful technique to decompose non-stationary signals with variable spectral characteristics over time. Using this technique, the fetal cardiac DUS signal is first decomposed into different scales with corresponding resolution levels. From the results, it is clear that the multiresolution wavelet analysis enabled the frequency contents of the fetal cardiac Doppler signals to be linked to the opening (o) and closing (c) of the fetal heart valves [aortic (A) and mitral (M)]. This technique was tested on DUS signal samples at a gestational age of 28–36 weeks. The valve movements were visualized as peaks in the detailed DUS signal at level 2 wavelet decomposition. The next stage is to assign each peak manually to the opening and closing of the cardiac valves.

### Blind Source Separation (BSS) Method

The BSS method was used to extract the fetal ECG from the abdominal ECG mixture, because the abdominal ECG is noisy and observing the fetal cardiac R wave is very difficult. Moreover, the correlation of the fetal cardiac cycle R–R interval with the interval of the R wave to each valve motion was examined, which has potential clinical applications. This correlation was introduced as a criterion for diagnosing fetal cardiac abnormalities. Previous researchers have investigated the automatic identification of these abnormalities as stated above (Sato et al., 2007; Khandoker et al., 2009b, 2010; Marzbanrad et al., 2014).

A non-invasive and automated method using an integrated fetal transabdominal ECG system and Doppler cardiogram was developed for the identification of fetal cardiac abnormalities (Khandoker et al., 2010). The authors used both the multiresolution wavelet analysis method and the Jensen–Shannon divergence method for the identification of the frequency contents of the Doppler signals for subsequent linking to the opening and closing of the fetal heart valves (aortic and mitral). Until recently, these cardiac time intervals have mostly been measured by ultrasound with manual identification of the fetal cardiac valve motion events. The potential for automated non-invasive assessment without obstetric ultrasonography expertise allows the development of integrated fetal ECG and cardiac Doppler signals from heart valve and wall motion events. Various applications of this technology may be feasible, enabling assessment of its value in antenatal monitoring of the fetal heart well-being (Khandoker et al., 2010; Marzbanrad et al., 2015a).

### Automated Estimation of Fetal Cardiac Timing Events from the DUS Signal Using the Hybrid Support Vector Machine (SVM)/Hidden Markov Model (HMM) Model

An automated methodology has been proposed to identify the occurrence of the cardiac events based on the sequence of the movements, timings, and patterns of both the valve and the wall in the fetal DUS signal components. These researchers proposed using the empirical mode decomposition (EMD) instead of STFT or wavelet analysis. This technique has been widely used for many applications, such as image processing, speech processing, and biomedical signal processing. The authors introduced three approaches to be combined with the EMD method for automated identification: the HMM, SVM, and hybrid SVM/HMM. In this case, the changes of the cardiac intervals were evaluated from the 16th to the 41st week of gestation (Echeverria et al., 2010; Mijovic et al., 2010; Marzbanrad et al., 2014, 2015a; Springer et al., 2016).

By comparing the results by Marzbanrad et al. (2014) with the pulsed Doppler image by Khandoker et al. (2009a), it can be concluded that the proposed hybrid algorithm afforded better results in terms of identification of the cardiac events. Moreover, compared to the conventional manual identification method by Khandoker et al. (2009a), a higher percentage of the fetal cardiac valve movement events was identified. In the study by Marzbanrad et al. (2014), the FECG was used as a reference for segmentation to facilitate the estimation of the timing of cardiac events. The proposed algorithm results provide a continuous and beat-to-beat identification of the fetal cardiac intervals, which can be used later for clinical purposes. Moreover, the authors noted a limitation in the proposed algorithm, in that a quantitative comparison with the pulsed-wave Doppler image-based valve motion timings was not provided. The more accurate method of trans-vaginal pulsed Doppler imaging can be used for fetuses in the first trimester. However, the authors claimed that the proposed method is compatible with wide continuous FHR monitoring during the second to third trimesters. The authors also stated that the need for more precise quantitative comparison of the proposed method results with pulsed Doppler images will require advanced research, such as image processing and recognition processes, which are beyond the scope of this study. Moreover, the authors suggested using the quantitative comparison in future studies (Kikallio et al., 2005; Marzbanrad et al., 2014).

### Identifying the Cardiac Events Automatically Using Machine Learning Techniques

In the conventional technique for identifying fetal cardiac events, the fetal cardiac timing events were manually assigned to the signal peaks and the time intervals were calculated. According to a previous study (Marzbanrad et al., 2014), the aim is to identify the cardiac events automatically using machine learning techniques. In this regard, each peak in the DUS signal should be classified as an indicator of one of the fetal cardiac valve timing events or none of them. The aforementioned researchers (Marzbanrad et al., 2014) used the HMM; this statistical model can allow the fetal cardiac events to be determined based on the probabilistic model of their occurrence sequence and timings in the DUS signal. However, the authors claimed that the amplitude as well as the timing of the peaks can also be used for the event classification.

Moreover, the authors used a powerful classifier, namely, SVM, for the classification of the fetal cardiac events. The authors noted certain limitations of SVM, including not considering the temporal dependence of the occurrence of events, faults in peak classification in some cardiac cycles, or an incorrect order of cardiac events. Furthermore, they proposed the hybrid HMM-SVM method as a solution to overcome the limitations of SVM and HMM. Each fetal cardiac cycle was segmented with reference to the FECG. The results of the proposed method showed the following achievements: 94.5% of mitral opening, 91.1% of mitral closing, 95.3% of aortic valve opening, and 98.8% of aortic valve closing. The authors analyzed the changes of the fetal cardiac intervals for three fetal age groups: 16–29, 30–35, and 36–41 weeks. These timings were identified using the proposed hybrid HMM-SVM technique, which provided more accurate results than the conventional manual approaches. The pulsed Doppler images were then used to verify the identified timings.

The K-means clustering method was also used to find the fetal DUS cardiac component patterns and match the DUS components of each cardiac cycle beat-to-beat to one of the models. To decompose the non-stationary signals with variable spectral characteristics over time, the authors proposed a multiresolution wavelet analysis method to apply to the DUS signal. The fetal cardiac valve movements were visualized as peaks in the detailed signal by using the wavelet analysis at level 2 wavelet decomposition. Subsequently, the hybrid SVM-HMM was used to identify the fetal cardiac valve motion events from the peaks of the DUS component and the model was trained specifically for its corresponding cluster (Marzbanrad et al., 2016).

### Hybrid EMD-Kurtosis Method

A new method has been proposed to estimate FHR and its variability from fetal DUS based on EMD and kurtosis statistics (Al-Angari et al., 2017). This method relies on computing the kurtosis function on the intrinsic mode functions extracted from the DUS signal to estimate cardiac beat-to-beat intervals. The authors also provided a comparison between the estimated beat-to-beat intervals using the proposed method and the autocorrelation function (AF) with respect to the R–R intervals computed from FECG. This method was tested on DUS signals from 44 pregnant mothers in the early (20 cases) and late (24 cases) gestational weeks. The authors reported that the EMD-kurtosis method showed superior performance for estimating the mean beat-to-beat intervals, with an average difference of 1.6 ms from the true mean R–R intervals, compared with the value of 19.3 ms obtained using the AF method. The EMD-kurtosis method is more robust than AF for low SNR cases and can be used in a hybrid system to estimate the beat-to-beat intervals from the fetal DUS signal. A limitation of this method is that the collected data were 1 min in length, which is insufficient to control the fetal states. This method can be easily implemented in a microprocessor in the same DUS machine or in a separate device for practical clinical use (The Society for Research in Child Development, 2015; Apostolidis and Hadjileontiadis, 2016).

In this study, the authors detected six different patterns for the DUS component. Two major findings were obtained. First, the generated patterns 1 and 6 occurred with significantly higher rates for the age group after 36 weeks than for the age group before 32 weeks. Second, the remaining patterns 3, 4, and 5 were observed with significantly higher rates for the early gestation group, whereas the percentage difference between the two age groups was not significant for pattern 2. The authors claimed that by comparing the proposed method with the technique in which clustering was not performed, the former allowed better precision and recall to be achieved. It was reported that the occurrence rates of five of the cardiac patterns differed between the fetuses older than 36 weeks and those under 32 weeks. Moreover, each cardiac pattern exhibited its own characteristics, such as amplitude range and timing of the peaks linked to the aortic and mitral valve motion. The authors compared the clustering method results to improve the identification of opening and closing of the mitral valve by the SVM-HMM method to the method without clustering, as verified using pulsed-wave Doppler images. The average precision and recall of the proposed technique with clustering were higher than those of the method without clustering by 83.4 and 84.2%, respectively (Marzbanrad et al., 2016).

In this review paper, several techniques have been discussed to address the challenges in fetal cardiac Doppler signal processing. These techniques facilitate the DUS signal analysis and classification, linear decomposition, and automated identification of the fetal cardiac events using machine learning techniques. Multiresolution wavelet analysis and BSS are techniques used to decompose non-stationary signals with variable spectral characteristics over time. This analysis has enabled the frequency contents of the fetal cardiac Doppler signals to be linked to the opening (o) and closing (c) of the fetal heart valves (aortic and mitral). These techniques have not yet been automated in terms of assigning each peak to the opening and closing of the cardiac valves.

Conventionally, fetal cardiac timing events are manually assigned to the signal peaks and the time intervals are calculated. This process requires experts and consumes time. The latest approach in fetal Doppler signal processing is the automated identification of the occurrence of the cardiac events based on the sequence of the movements, timings, and patterns of both the valve and the wall in the fetal DUS signal components. This technique consists of three approaches to be combined with the EMD method for automated identification: the HMM, SVM, and the hybrid SVM/HMM method. The technique was used to analyze the changes in the fetal cardiac intervals for three fetal age groups: 16–29, 30–35, and 36–41 weeks. Although this technique led to an improvement in the estimation of fetal cardiac timing events, more precise quantitative comparison of the results is required. In addition, the K-means clustering method has been used to ascertain the fetal DUS cardiac component patterns and match the DUS components of each cardiac cycle “beat-to-beat” to one of the models. Six different patterns for the DUS component were found. The use of the clustering method improved the identification of the opening and closing of the mitral valve by the SVM-HMM method compared with the method without clustering. The key limitation of the current automated estimation of fetal cardiac intervals from DUS signals is the complexity of these systems.

## Future Work

Several issues concerning fetal cardiac Doppler signal processing still need to be resolved. First, one limitation of the latest new technique for computerized estimation of fetal cardiac time intervals from DUS signals is that the quantitative comparison with the pulsed-wave Doppler image-based valve motion timings has not yet been achieved (Marzbanrad et al., 2015a). Trans-vaginal pulsed Doppler imaging is considered one of the most accurate techniques, yet it can only be performed in the first trimester. The latest computerized estimation of fetal cardiac time intervals from the fetal cardiac DUS signal will help in continuing with the same accuracy during the remainder of the pregnancy. As for future studies, previous researchers suggested the application of image recognition processes on the pulsed Doppler images to enhance the quantitative comparison of the result accuracy (Marzbanrad et al., 2014).

In order to measure the correctness of the automated identification of the fetal cardiac valve motion by hybrid SVM-HMM methods, M-mode and pulsed-wave Doppler images were used (Marzbanrad et al., 2014, 2016). The findings suggested that more quantitative comparisons should be performed in future investigations. As the fetus grows in the late gestation period, its movement increases, which results in the non-stationary nature of the fetal cardiac Doppler signal. Longer fetal cardiac Doppler recordings are required to evaluate the non-stationary nature and improve the FHR accuracy. The advanced analysis of the fetal cardiac non-stationary Doppler signal and its influence on the FHR monitoring remains a topic for future studies (Marzbanrad et al., 2015b). The variation of cardiac features is a limitation for the predefined range of the valve motions in the proposed method (Marzbanrad et al., 2015a). The recommendation of these researchers for future studies is to assess the validity of the measurements on the abnormal cases. Finally, reducing the complexity of the current automated estimation of fetal cardiac intervals from DUS signals should be subject to future investigation.

## Conclusion

The current survey has discussed the importance of clinical FHR monitoring and provided a critical overview of the existing techniques. Several solutions have been proposed to overcome the effect of the non-stationary nature of the fetal cardiac Doppler signal on a beat-to-beat basis, in addition to the variation of the spectral characteristics and signal content over time. These solutions include band-pass filters, STFT, and wavelet analysis. With respect to DUS signal analysis and classification, linear decomposition techniques such as BSS for source separation have shown promising results in the isolation of fetal cardiac Doppler signal components. Furthermore, several automated techniques for the identification of valve movements using fetal cardiac Doppler signals have been discussed to overcome the shortcomings of manual techniques, including their time-consuming nature, and to increase the FHR measurement accuracy. It is important to mention that using machine learning techniques within the context of FHR monitoring can potentially open up new research areas that have so far gone unexplored. Furthermore, potential directions for future research have been suggested to improve current fetal cardiac Doppler signal processing and analysis techniques. Finally, the growing interest in fetal cardiac DUS is set to provide new opportunities for reliable and accurate FHR monitoring.

## Author Contributions

SA is the first author who is a PhD student, AK is the main supervisor, and SJ is the secondary supervisor.

## Conflict of Interest Statement

The authors declare that the research was conducted in the absence of any commercial or financial relationships that could be construed as a potential conflict of interest.
