# Supplementary material for: Fetal Cardiac Doppler Signal Processing Techniques: Challenges and Future Research Directions
Source: Front Bioeng Biotechnol. 2017 Dec 22;5:82. doi: 10.3389/fbioe.2017.00082 (PMC5743703; doi:10.3389/fbioe.2017.00082)
Supplement: Supplementary file 1 [file table_1.docx]

Supplementary Material

A Review of Fetal cardiac Doppler Signal Processing for Screening Foetal Well Being

Saeed Alnuaimi^1^, Shihab Jimaa, Ahsan H. Khandoker

Corresponding Author: saeed.alnuaimi@kustar.ac.ae

# Supplementary Figures and Tables

## Supplementary Figures


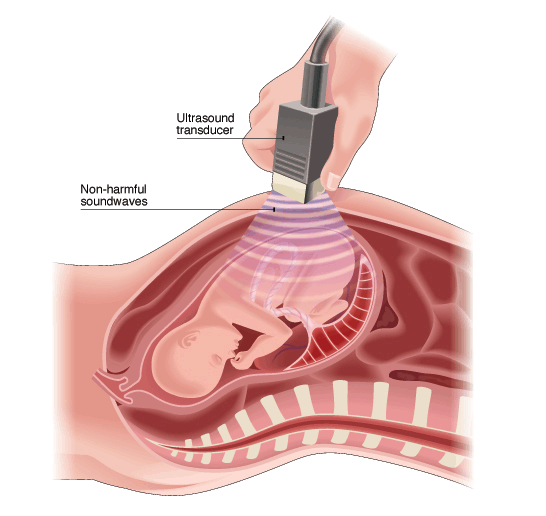

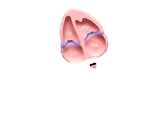


Figure 1. Fetal cardiac activity assessment using Doppler ultrasound [BBC].

**Figure 2: Simultaneously captured DUS and fetal ECG signals (period 2.5 seconds). Arrows pointing the high-frequency component of the DUS signal.**

Figure 3. Available Fetal cardiac Doppler Literature in IEEE, Science Direct & PubMed Databases.


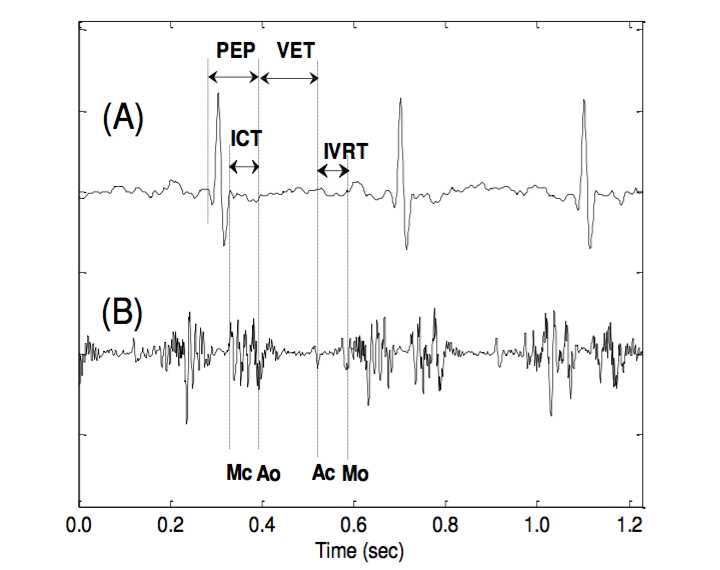


Figure 2. Example of simultaneously recorded fetal ECG and Doppler ultrasound data. (A) fetal ECG signal (B) Fetal heart Doppler signal (1).

## Supplementary Tables

Table 1: Mean ± standard error of the average time intervals (msec) over 45 normal fetuses and the accuracy of identified events.

|  | Reference | | 2014 | |
| --- | --- | --- | --- | --- |
| Intervals | Mean ± Standard Error | Rate | Mean ± Standard Error | Rate |
| R-R | 421 ± 33 | 100% | 413.6 ± 26 | 100% |
| R-Mc | 27.7 ± 9.4 | 84.4% | 14.3 ± 2.3 | 91.1% |
| R-Ao | 79.3 ± 17.4 | 87.0% | 51.1 ± 3.4 | 95.3% |
| R-Ac | 224.7 ± 13.3 | 97.6% | 204.6 ± 5.5 | 98.8% |
| R-Mo | 294.6 ± 21.3 | 89.70% | 276.4 ± 5.4 | 94.5% |
| Ao-Ac | 144 ± 26.7 | 87.0% | 153.5 ± 6.3 | 94.6% |

Table 2: The timing intervals records of the normal fetuses and fetuses with heart abnormalities.

|  | Normal fetuses | Abnormal fetuses |
| --- | --- | --- |
| PEP (Pre-ejection period) | 75.0±11.9 (msec) | 89.0±10.3 (msec) |
| VET (Ventricular ejection time) | 153.2±18.9 (msec) | 168.6±25.0 (msec) |
| ICT (Isovolumic contraction time) | 50.0±15.9 (msec) | 52.2±17.2 (msec) |
| IVRT (Isovolumic relaxation time) | 69.6±9.7 (msec) | 51.6±13.7 (msec) |

Table 3: Comparison of previous filtering and measurement frequencies in the literature.

| Author, Year (Reference) | Title | Objective & Proposed method design overview | Time-frequency analysis techniques | US freq. (MHz) | Filtering frequencies (Hz) |
| --- | --- | --- | --- | --- | --- |
| (2) | The information content of Doppler ultrasound signals from the fetal heart | - Improving the performance of fetal heart rate (FHR) monitors. - Instrumentation was constructed to enable the simultaneous collection of Doppler audio signals and the trans-abdominal fetal ECG (for signal registration), with a total of 22 recordings being made with an average length of around 20 minutes. | Short-time Fourier transform (STFT) | 1.5 | Generates 600 & 300 Hz (valve & wall) |
| (3) | Systolic Time Intervals of the Fetal Cardiac | - Study systolic Time Intervals of the Fetal Cardiac, | Band-pass filter | 2 | 600–2000 Hz for valve motion |
| (4) | De-noising of the Doppler fetal heart rate signal with wavelet threshold filtering based on spatial correlation. | - Analyzes the characteristics of Doppler echo signal, using the spatial correlation to estimate original noise variance, educes the de-noising threshold in every scale, and then develops an adaptive method calculating de-noising threshold, finally makes certain amendments for it in practice. | wavelet decomposition algorithm | 2.75 | 1350–2750 Hz for cardiac activity |
| (5) | Comparison of Doppler ultrasound and direct electrocardiography acquisition techniques for quantification of fetal heart rate variability | - Evaluate the commonly used Doppler ultrasound technique for monitoring of mechanical activity of fetal heart. - Accuracy of beat-to-beat interval determination together with its influence on indices describing the fetal heart rate (FHR) variability calculated automatically using computer-aided fetal monitoring system were examined. | Autocorrelation technique | 1 | 200–1000 Hz for cardiac activity |
| (6) | Using modified fetal monitor and signal processing to detect fetal breathing movement | - Propose a simple technique to automatically detect the fetal breathing movements (FBM) from a directional Doppler signal. | The Doppler signal was filtered with an analog band-pass filter between 5 and 200 Hz. | 2.3 | 5–60 Hz for fetal movement |
| (7) | Computerized analysis of cardiotocograms and fetal movements | - The amplitude and interval of the electrical deflections produced by fetal movement were analyzed. - Fetal behavioral states were automatically recognized in this way. - Cross-correlational analysis of fetal heart rate and fetal movement showed a close relationship between the two phenomena. | Cross-correlational analysis | 2 | 20–80 Hz for fetal movement |
| (8) | Coping with limitations of Doppler ultrasound fetal heart rate monitors | - Considering that computerised analysis of the acquired FHR data should work with today’s fetal monitor, procedures to cope with information deficiency are proposed. | Band-pass filter | 2 | 150–350 Hz for cardiac activity |
| (9) | Method and Apparatus for Calculating the Fetal Heart Rate | - Parallel processing of the demodulated ultrasound signal in various pass-bands, for which purpose the signal is fed to filters of different frequency characteristics. The out put of the pass band with the better result is used for fetal heart rate circulation. | Highly sophisticated autocorrelation technique | 1 | 100–500 Hz for cardiac activity |
| (10) | A test and simulation device for Doppler-based fetal heart rate monitoring. | - Design a reliable system that will be used to test Doppler FHR monitors. - This device generates a similar Doppler frequency shift of fetal cardiac activity including the heart’s wall and valve motions. | Band-pass filter | 1 | Simulates valve & wall motion generating 612 Hz & 212 Hz |
| (11) | Model-Based Estimation of Aortic and Mitral Valves Opening and Closing Timings in Developing Human Fetuses | - An efficient model is proposed using K-means clustering and hybrid Support Vector Machine–Hidden Markov Model (SVM–HMM) modeling techniques. - Opening and closing of the cardiac valves were detected from peaks in the high frequency component of the DUS signal decomposed by wavelet analysis. | The multi-resolution Wavelet analysis | 1.5 | 1–100 Hz finite impulse response filter. |
| (12) | Automated Estimation of Fetal Cardiac Timing Events From Doppler Ultrasound Signal Using Hybrid Models | - A new noninvasive method is proposed for automated estimation of fetal cardiac intervals from Doppler Ultrasound (DUS) signal. - This method is based on a novel combination of empirical mode decomposition (EMD) and hybrid support vector machines—hidden Markov models (SVM/HMM). - EMD was used for feature extraction by decomposing the DUS signal into different components (IMFs), one of which is linked to the cardiac valve motions. | Empirical Mode Decomposition | 1.15 | 100 Hz |
| (13) | Logistic Regression-HSMM-Based Heart Sound Segmentation | - Addresses the problem of the accurate segmentation of the first and second heart sound within noisy real-world PCG recordings using an HSMM, extended with the use of logistic regression for emission probability estimation. - Implementing a modified Viterbi algorithm for decoding the most likely sequence of states, and evaluated this method on a large dataset. | Wavelet Envelope | 44.1 kHz | Between 65 and 85 Hz |

1. A. H. Khandoker, Y. Kimura, M. Palaniswami and S. Marusic: Identifying fetal heart anomalies using fetal ecg and doppler cardiogram signals (2010)

2. J. A. C. S. A. Shakespeare, B.R. Hayes-Gill, K. Bhogal, D. K. James The information content of doppler ultrasound signals from the fetal heart (2001)

3. M. a. C. B. M. J. Yuji Murata, MD, FACOG: Systolic Time Intervals of the Fetal Cardiac (1974)

4. P. L. X. Yang, X. Zhang, Z. Bian and B. Wang: De-noising of the Doppler fetal heart rate signal with wavelet threshold filtering based on spatial correlation. *The 1st International Conference on Bioinformatics and Biomedical Engineering (ICBBE 2007)*, 928-931 (2007)

5. J. W. a. K. H. J. Jezewski: Comparison of Doppler Ultrasound and Direct Electrocardiography Acquisition Techniques for Quantification of Fetal Heart Rate Variability. *IEEE Transactions on Biomedical Engineering*, 53(5), 855 - 864 (2006) doi:10.1109/TBME.2005.863945

6. K. B. Foulqiere K, Vilgbergsson G, Berson M: Using modified fetal monitor and signal processing to detect fetal breathing movement. *IEEE 2000 Ultrasonics Symposium*, 1391–1394 (2000)

7. K. MAEDA: Computerized analysis of cardiotocograms and fetal movements. *Baillière's Clinical Obstetrics And Gynaecology*, 4, 797-813 (1990)

8. W. J. Jezewski J, Horoba K, Graczyk S, Gacek A.: Coping with limitations of Doppler ultrasound fetal heart rate monitors. *IEEE 14th Conference of the Biomedical Engineering Society of India* (1995)

9. S. M. Boss A: Method and Apparatus for Calculating the Fetal Heart Rate. In: Ed P. H.-P. Company. USA (1992)

10. A. Mert, M. Sezdİ and A. Akan: A test and simulation device for Doppler-based fetal heart rate monitoring. *Turkish Journal of Electrical Engineering & Computer Sciences*, 23, 1187-1194 (2015) doi:10.3906/elk-1306-224

11. F. Marzbanrad, Y. Kimura, K. Funamoto, S. Oshio, M. Endo, N. Sato, M. Palaniswami and A. H. Khandoker: Model-Based Estimation of Aortic and Mitral Valves Opening and Closing Timings in Developing Human Fetuses. *IEEE J Biomed Health Inform*, 20(1), 240-8 (2016) doi:10.1109/JBHI.2014.2363452

12. F. Marzbanrad, Y. Kimura, K. Funamoto, R. Sugibayashi, M. Endo, T. Ito, M. Palaniswami and A. H. Khandoker: Automated estimation of fetal cardiac timing events from Doppler ultrasound signal using hybrid models. *IEEE J Biomed Health Inform*, 18(4), 1169-77 (2014) doi:10.1109/JBHI.2013.2286155

13. D. B. Springer, L. Tarassenko and G. D. Clifford: Logistic Regression-HSMM-Based Heart Sound Segmentation. *IEEE Trans Biomed Eng*, 63(4), 822-32 (2016) doi:10.1109/TBME.2015.2475278
